# Lung involvement percentage in patients with COVID-19 during the Omicron wave in China: a SHAP-explained machine learning study from a single center

**DOI:** 10.3389/fpubh.2025.1728282

**Published:** 2026-01-15

**Authors:** Yuhang Ma, Li Ye, Jing Pan, Dongyuan Shen, Qiang Wang, Bin Song, Yiliang Shen, Xiaoqiang Zhu, Feng Chen, Jian Shi, Qin Ye, Siwei Qin, Rong Ren, Xin Luo, Jun Xu, Jianzhong Zhao, Dongxing Zhu, Qiujuan Zhou, Yiming Zhu, Biquan Zhang

**Affiliations:** Department of Radiology, Suzhou Hospital of Integrated Traditional Chinese and Western Medicine, Suzhou, China

**Keywords:** advanced age, COVID-19, daily case counts, lung involvement percentage, machine learning, Omicron, outbreak, SHAP

## Abstract

**Background:**

Following the lifting of China’s stringent lockdown policy on December 7, 2022, COVID-19 cases surged in a pattern, creating unprecedented strain on healthcare systems. The Omicron variant, characterized by high transmissibility and rapid spread, led to a sharp rise in infections. Understanding its clinical impact—particularly on lung involvement percentage—is crucial for optimizing patient care under such outbreak conditions. This study aimed to assess the extent of lung involvement percentage during the outbreak and its major associations.

**Methods:**

The hospital’s daily computed tomography examination volume was quantified using artificial intelligence–based pulmonary inflammation analysis software and used as an indicator of epidemic intensity. Associations between lung involvement percentage and age, sex, and daily case counts were evaluated using GEE Logistic Regression, complemented by machine learning models. Model interpretation was performed using SHapley Additive exPlanations.

**Results:**

GEE Logistic regression demonstrated that age was strongly associated with lung involvement (OR 1.0813, 95% CI 1.0703–1.0925, *p* < 0.0001), while daily case counts also showed a small but significant independent association (OR 1.0033, 95% CI 1.0018–1.0047, *p* < 0.0001). Sex exhibited only minimal association (OR 0.8098, 95% CI 0.6983–0.9391, *p* = 0.0053). Complementary machine learning analyses, including gradient boosting, identified age as the dominant contributor, followed by daily case counts with a small effect and sex with minimal contribution. SHAP analysis provided interpretable insights into how each feature influenced model predictions at both global and individual levels.

**Conclusion:**

During the Omicron surge, greater age and higher daily case counts were associated with higher lung involvement percentage. These associations highlight the relevance of demographic and epidemic factors in characterizing pulmonary findings during large-scale outbreaks.

## Introduction

Since the emergence of coronavirus disease 2019 (COVID-19), caused by severe acute respiratory syndrome coronavirus 2 (SARS-CoV-2), the pandemic has posed a significant global health challenge. After multiple variant-driven waves worldwide, China lifted stringent lockdown measures in China on December 7, 2022, Omicron’s high transmissibility lead to a rapid increase in infections, exerting immense pressure on healthcare systems. Research to date has mainly explored the COVID-19 epidemiology and clinical features, as well as factors such as age, gender, and case counts.

### Age-related characteristics of COVID-19

Age is a critical predictor of COVID-19 severity and mortality. Previous studies have shown that increasing age is closely associated with declining immune function and the accumulation of underlying comorbidities, making older patients more susceptible to developing severe disease (1). However, in clinical practice, relying solely on age to assess individual risk may result in misclassification, potentially affecting resource allocation and patient management.

### Sex as a stratification factor

Numerous studies have demonstrated significant differences between males and females in clinical presentation and outcomes following SARS-CoV-2 infection, which may be attributed to sex-related variations in immune responses, hormone levels, and behavioral factors (2). Males generally exhibit lower innate and adaptive immune responses, including reduced CD4^+^ T cell counts, diminished CD8^+^ T cell cytotoxic activity, and decreased immunoglobulin production by B cells (3). Moreover, physiological responses to viral infections differ between sexes, with reports indicating that the immune system of females is approximately twice as robust as that of males (4). Sex-related differences in immune response efficiency are associated with disease outcomes, with males exhibiting a significantly higher risk of COVID-19–related mortality than females (hazard ratio 1.59) (5). Some researchers have linked these findings to genes located on the X chromosome (6). Estrogen can enhance the immunomodulatory activity of vitamin D, thereby improving infection outcomes (7). In contrast, male sex hormones may increase susceptibility to COVID-19 and worsen disease prognosis. First, they are thought to facilitate viral entry by upregulating angiotensin-converting enzyme 2 (ACE2) receptors, the entry point for SARS-CoV-2. Second, testosterone exhibits immunosuppressive effects, potentially dampening antibody responses. Males may benefit from T cell immune stimulants and anti-testosterone interventions, while estrogen could be utilized to reduce COVID-19 disease severity (8). A study conducted in China reported that males and females had similar susceptibility to infection. However, independent of age, infected males faced poorer clinical outcomes and a higher risk of mortality (9).

### Impact of epidemic intensity on COVID-19 severity

During periods of strict lockdown in China, sporadic COVID-19 cases generally exhibited mild disease severity. In contrast, under non-lockdown conditions, the severity of cases appeared to increase more markedly than anticipated, suggesting that the intensity of viral spread may significantly influence disease outcomes. It is hypothesized that higher transmission rates lead to increased viral loads, making patients more susceptible to severe complications such as acute respiratory distress syndrome (ARDS) and multiple organ failure; however, direct evidence supporting this hypothesis remains limited. Additionally, during outbreak peaks, shortages of healthcare resources—such as hospital beds and ICU capacity—may further exacerbate mortality risk (10, 11).

Despite these advances, prior studies have largely been conducted under conditions of strict containment or during earlier, less transmissible SARS-CoV-2 variants. Consequently, evidence describing the relationship between age, sex, daily case counts, and CT-based lung involvement during a large-scale outbreak without lockdown remains limited. In particular, few studies have examined how epidemic intensity, measured by daily case counts, may influence radiographic severity in real-world settings where healthcare systems are under substantial pressure. To address these gaps, the present study aims to characterize the distribution of CT-determined lung involvement percentage and to evaluate its association with age, sex, and daily case counts.

## Materials and methods

### Study design

This study is a retrospective case series of consecutive patients who underwent chest CT during the Omicron outbreak in Suzhou. Machine learning–based predictive modeling was applied alongside classical statistical analyses to evaluate associations between lung involvement and key patient- and population-level factors.

### Setting

The study was conducted at Suzhou Hospital of Integrated Traditional Chinese and Western Medicine, a hospital serving a mixed urban–rural population. The abandonment of China’s zero-COVID policy on December 7, 2022, coincided with the spread of the highly transmissible Omicron variant. This combination led to a marked increase in daily CT volume during the late 2022 and early 2023.

### Participants

All consecutive chest CT scans performed at the hospital during the study period were screened. Inclusion criteria: (1) chest CT performed between December 14, 2022, and January 10, 2023; (2) exclusion of pneumonia caused by other infections; (3) only the first CT per patient was included.

### Variables

Outcome: Lung involvement percentage, defined as the percentage of lesioned lung, quantified using AI-based lung segementation.

Positivtiy of lung involvement, defined as a binary variable indicating the presence or absence of lung lesions, derived from lung involvement percentage using a predefined threshold.

Factor: Age, sex, and the daily case counts of chest CT examinations performed at the hospital.

### Data sources/measurement

Each case was labeled as positive based on radiologist report–derived findings for preliminary exploratory analysis. Lung involvement percentage was quantified using AI-based segmentation software, which automatically segmented lung parenchyma and generated a continuous measure of involved lung tissue for subsequent modeling. AI-derived lung involvement maps were independently reviewed by two radiologists to identify and exclude false-positive findings caused by respiratory motion artifacts, dependent atelectasis, and obvious chronic pulmonary lesions that could be misclassified as COVID-19 pneumonia.

### Statistical methods

Associations were examined using generalized estimating equations (GEE) logistic regression. To address potential correlation among patients scanned on the same day, we specified an exchangeable working correlation structure and treated scanning day as the clustering variable. This approach provides consistent estimates of regression coefficients while adjusting standard errors for within-cluster correlation. Odds ratios (ORs) with 95% confidence intervals (CIs) were derived from model coefficients to quantify the strength and uncertainty of associations.

For modeling purposes, the continuous lung involvement percentage was converted into a binary outcome using a threshold optimized based on the balance between F1 score, precision, and recall. Predictive modeling was performed with logistic regression (LR), support vector machine (SVM), random forest (RF), gradient boosting (GB), decision tree (DT), naïve Bayes (NB), k-nearest neighbors (KNN), and multilayer perceptron (MLP) models. All models were trained and evaluated using grouped 5-fold cross-validation, with grouping defined by scanning day to prevent information leakage across folds. Model interpretation was conducted using SHapley Additive exPlanations (SHAP). Performance metrics included accuracy (ACC), F1 score, area under the receiver operating characteristic curve (ROC AUC), and area under the precision–recall curve (PR AUC). Differences before and after adding daily case counts were tested by the DeLong and Z tests. A *p*-value < 0.05 was considered statistically significant.

## Results

### Participants

**Figure 1:**
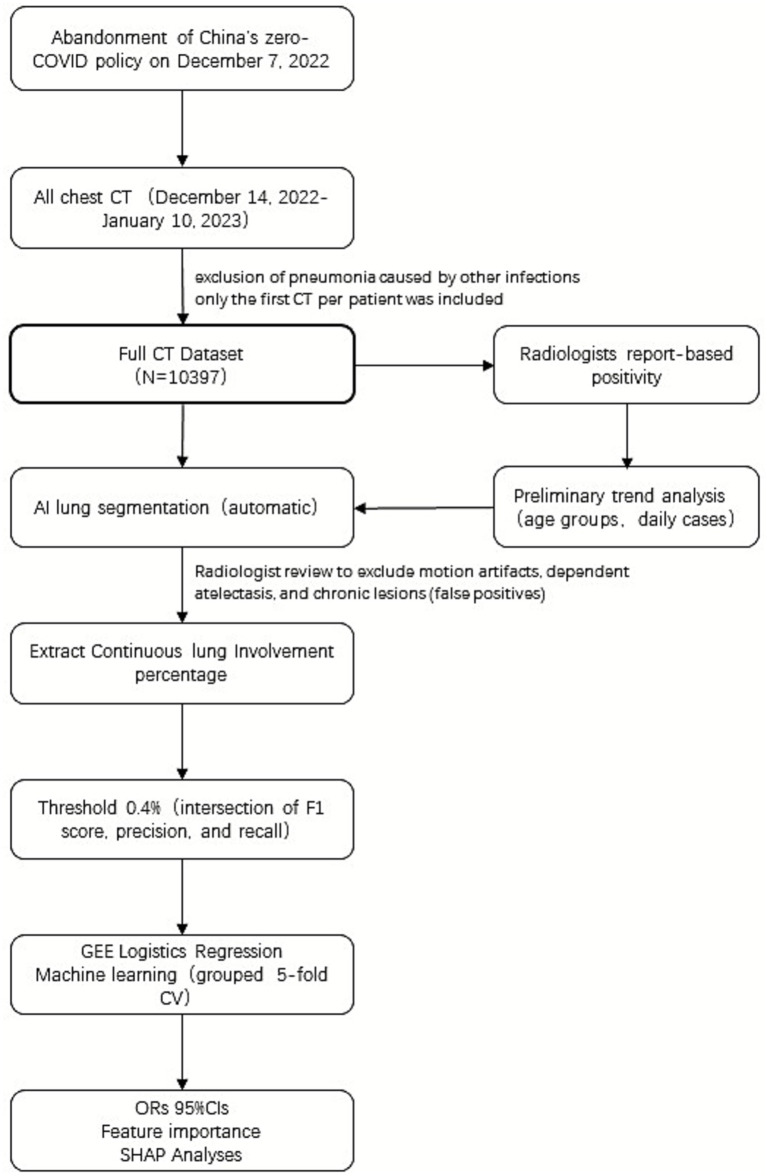
Flow diagram of data selection and analyses.

After applying inclusion and exclusion criteria (see flow diagram, Figure 1), 10,397 unique patients were included in the analysis. The cohort comprised 4,630 males and 5,767 females, with mean age 48.96 ± 18.91 years.

### Characteristics and lung involvement percentage

**Figure 2:**
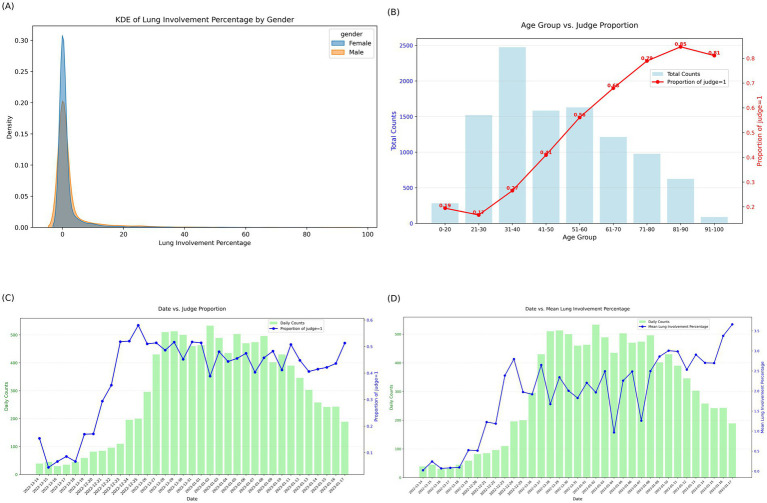
**(A)** Kernel density estimation (KDE) of lung involvement percentage by sex. The distribution of lung involvement percentage is shown separately for male and female patients. All genders exhibit a right-skewed distribution, with the majority of cases showing minimal lung involvement. **(B)** Age group and proportion of lung involvement (judge = 1). Bar plots represent the total number of patients in each age group, while the red line indicates the proportion of cases with lung involvement (judge = 1). The proportion of lung involvement increases sharply with age, particularly after 50 years old, suggesting that age is a strong risk factor for lung involvement. **(C)** Temporal trend of daily counts and proportion of lung involvement (judge = 1). The green bars indicate the daily counts, and the blue line shows the proportion of cases with lung involvement (judge = 1) over time. The proportion of affected cases increased rapidly during the early outbreak phase and remained high during the epidemic peak. **(D)** Temporal trend of Mean lung involvement percentage and daily counts. The green bars represent daily counts, while the blue line indicates the mean lung involvement percentage for that date. The Mean lung involvement percentage increased progressively with rising daily counts.

Characteristics are summarized in Table 1. Kernel density estimation plots of lung involvement percentage stratified by sex (Figure 2A) show that most patients had minimal lung involvement, with a small proportion exhibiting higher percentages, indicating a right-skewed distribution.

**Table 1:** Characteristics of all patients.

Variable	Total (*N* = 10,397)	Survivors (*N* = 10,381)	Deaths (*N* = 16)	Deaths (%)	*p*-value
Age (years, mean ± SD)	48.9 ± 18.9	48.9 ± 18.9	79.0 ± 10.0	0.15%	<0.0001
Age group 0–20, *n* (%)	283 (2.7%)	283 (2.7%)	0 (0.0%)	0.0%	
Age group 21–30, *n* (%)	1,521 (14.6%)	1,521(14.6%)	0 (0%)	0.0%	
Age group 31–40, *n* (%)	2,474 (23.8%)	2,474 (23.8%)	0 (0%)	0.0%	
Age group 41–50, *n* (%)	1,584 (15.2%)	1,584 (15.2%)	0 (0%)	0.0%	
Age group 51–60, *n* (%)	1,629 (15.7%)	1,628 (15.7%)	1 (6.25%)	0.06%	
Age group 61–70, *n* (%)	1,213 (11.7%)	1,212 (11.7%)	1 (6.25%)	0.06%	
Age group 71–80, *n* (%)	978 (9.4%)	973 (9.37%)	5 (32.25%)	0.51%	
Age group 80 + *n* (%)	715 (6.88%)	706 (6.8%)	9 (56.25%)	1.26%	
Female, *n* (%)	5,764 (55.5%)	5,759 (55.5%)	5 (31.2%)	0.09%	0.0893
Male, *n* (%)	4,627 (44.5%)	4,616 (44.5%)	11 (68.8%)	0.24%	
Lung involvement percentage (%, mean ± SD)	2.2 ± 7.1	2.1 ± 6.6	69.1 ± 14.5		<0.0001
Lung involvement percentage = 0, *n* (%)	6,175 (59.4%)	6,175 (59.4%)	0 (0.0%)		<0.0001

Age-stratified positivity proportions based on radiologist reports (Figure 2B) showed a progressively higher proportion of positive cases in older age groups. Date-stratified radiologist report–based positivity proportions (Figure 2C) exhibited temporal patterns characterized by higher positivity during periods with increased daily case counts. Similarly, the date-stratified mean lung involvement percentage (Figure 2D) showed a comparable temporal trend, with higher mean involvement observed on days with higher case counts.

### GEE Logistic regression analysis

GEE Logistic regression showed that age was strongly associated with lung involvement (OR 1.0813 per year increase, 95% CI 1.0703–1.0925, *p* < 0.0001), while daily case counts also showed a modest but significant association (OR 1.0033 per 1-case increase, 95% CI 1.0018–1.0047, *p* < 0.0001). Sex exhibited a minimal association, with females exhibiting lower odds of lung involvement compared with males (OR 0.8098, 95% CI 0.6983–0.9391, *p* = 0.0053).

### Predictive performance of machine learning models

**Figure 3:**
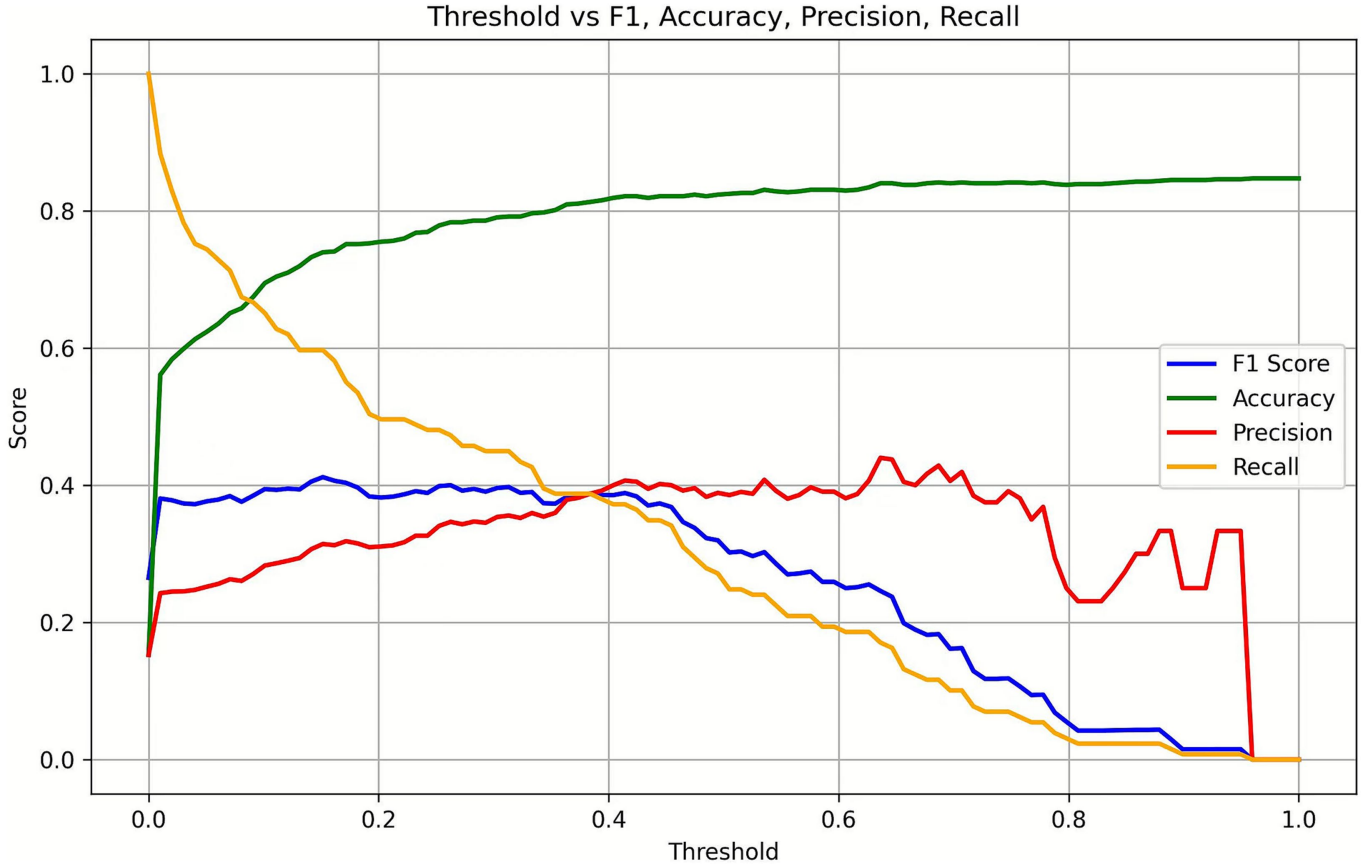
Threshold optimization based on F1 score, precision, and recall. F1 score, precision, and recall were plotted across different thresholds. The three metrics intersected around 0.4%, which was selected as the threshold for classification.

For predictive modeling, age, sex and the inclusion or exclusion of daily case counts were used as independent variables, while binary lung involvement percentage served as the dependent variable. The binary classification threshold (0.4%) was determined based on the intersection of F1 score, precision, and recall (Figure 3). ACC, F1 score, ROC AUC, and PR AUC are summarized in Table 2.

**Table 2:** Comparison of model performance before and after incorporating daily case counts.

Model	Condition	ACC	F1	ROC AUC	DeLong *p*-value	PR AUC	Z-test *p*-value
LR	Before	0.7894	0.5783	0.8228	Reference	0.5903	Reference
LR	After	0.7873	0.5865	0.8229	0.7640	0.5894	0.5020
SVM	Before	0.7860	0.5465	0.7868	Reference	0.5723	Reference
SVM	After	0.7876	0.5971	0.8208	0.0000	0.5903	0.0520
RF	Before	0.7863	0.5545	0.8145	Reference	0.5725	Reference
RF	After	0.7502	0.5061	0.7572	0.0000	0.4994	0.0000
GB	Before	0.7851	0.5449	0.8199	Reference	0.5769	Reference
GB	After	0.7811	0.5613	0.8180	0.3780	0.5684	0.2520
DT	Before	0.7832	0.5555	0.8127	Reference	0.5725	Reference
DT	After	0.7260	0.5127	0.7099	0.0000	0.5230	0.0000
KNN	Before	0.7486	0.4704	0.7057	Reference	0.4851	Reference
KNN	After	0.7570	0.5207	0.7593	0.0000	0.5212	0.0440
NB	Before	0.7870	0.5817	0.8230	Reference	0.5907	Reference
NB	After	0.7879	0.5917	0.8228	0.5900	0.5894	0.0400
MLP	Before	0.7860	0.5967	0.8228	Reference	0.5895	Reference
MLP	After	0.7792	0.5587	0.8147	0.0000	0.5714	0.0000

Performance metrics of eight machine learning models—including LR, SVM, RF, GB, DT, KNN, NB, and MLP—before and after inclusion of daily case counts as an additional variable. Model performance was evaluated using ACC, F1 score, ROC AUC, and PR AUC. Statistical differences were assessed using the DeLong test (for ROC AUC) and Z-test (for PR AUC). 1. “Before” indicates model performance using age and gender as predictors. 2. “After” indicates model performance after incorporating daily case counts. 3. “Reference” indicates the baseline value for comparison in statistical tests.

Across the eight evaluated models, logistic regression demonstrated consistently strong and stable performance both before and after the inclusion of daily case counts. In contrast, the impact of adding daily case counts varied across machine learning models. While modest improvements in discrimination were observed for certain models (e.g., SVM and KNN), several tree-based and neural network models exhibited a decline in performance under grouped five-fold cross-validation. DeLong tests identified statistically significant changes in ROC AUC for SVM, RF, DT, KNN and MLP, whereas PR AUC comparisons using Z-tests showed significant differences for most models.

**Figure 4:**
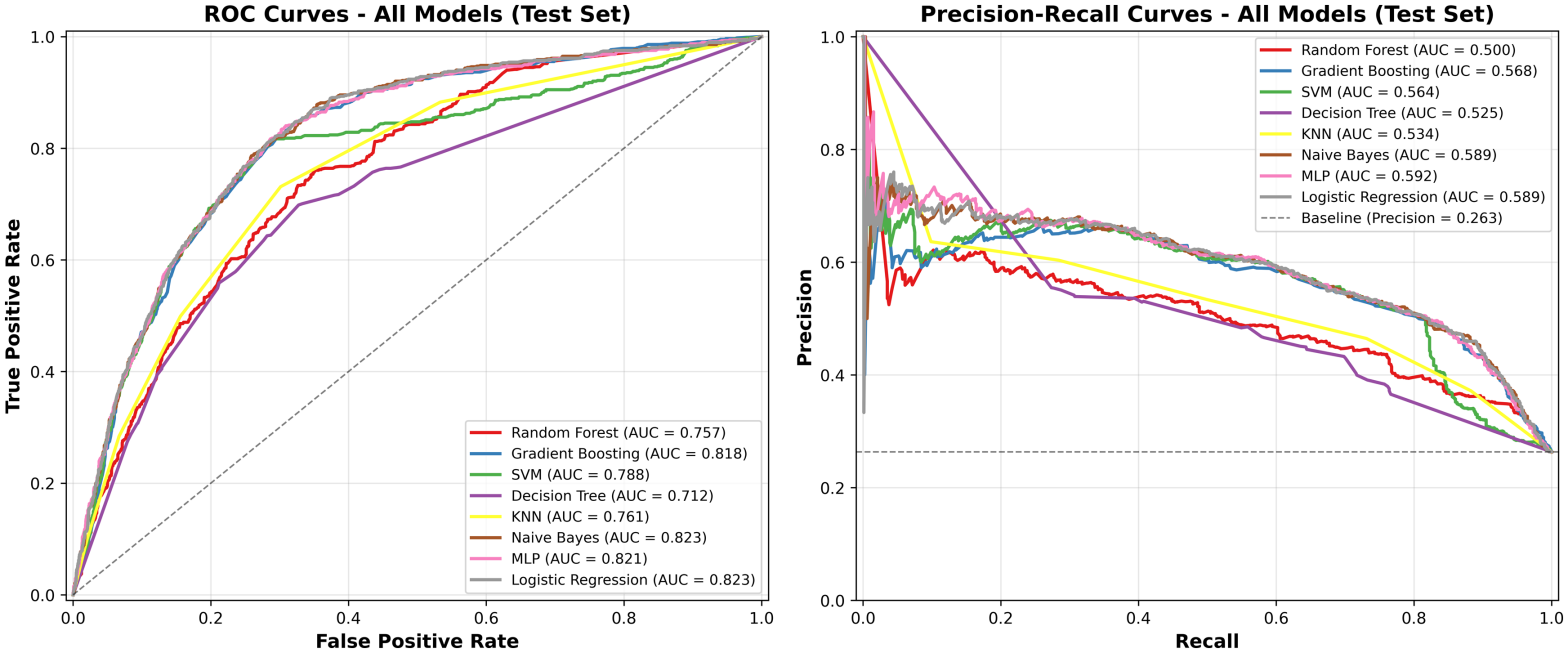
Performance comparison of machine learning models for predicting lung involvement percentage. **(A)** Receiver operating characteristic (ROC) curves. **(B)** Precision-recall (PR) curves. The LR and SVM model achieved the highest AUC in ROC (0.828) and PR (0.645) analyses, indicating superior overall performance.

**Figure 5:**
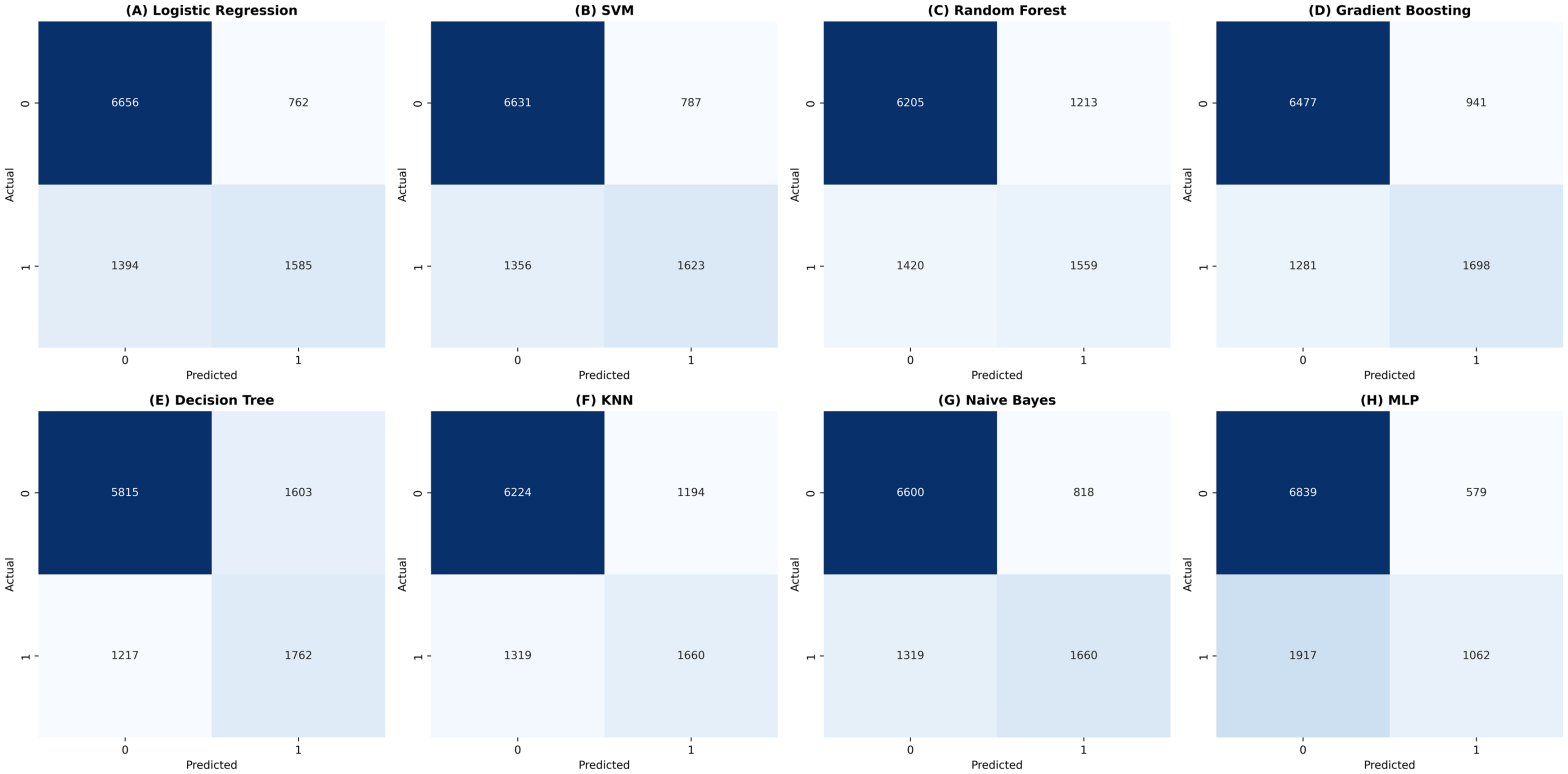
Confusion matrix heatmaps for eight classification models. Confusion matrices illustrate the classification performance of eight machine learning models in predicting lung involvement. Darker colors represent higher counts.

**Figure 6:**
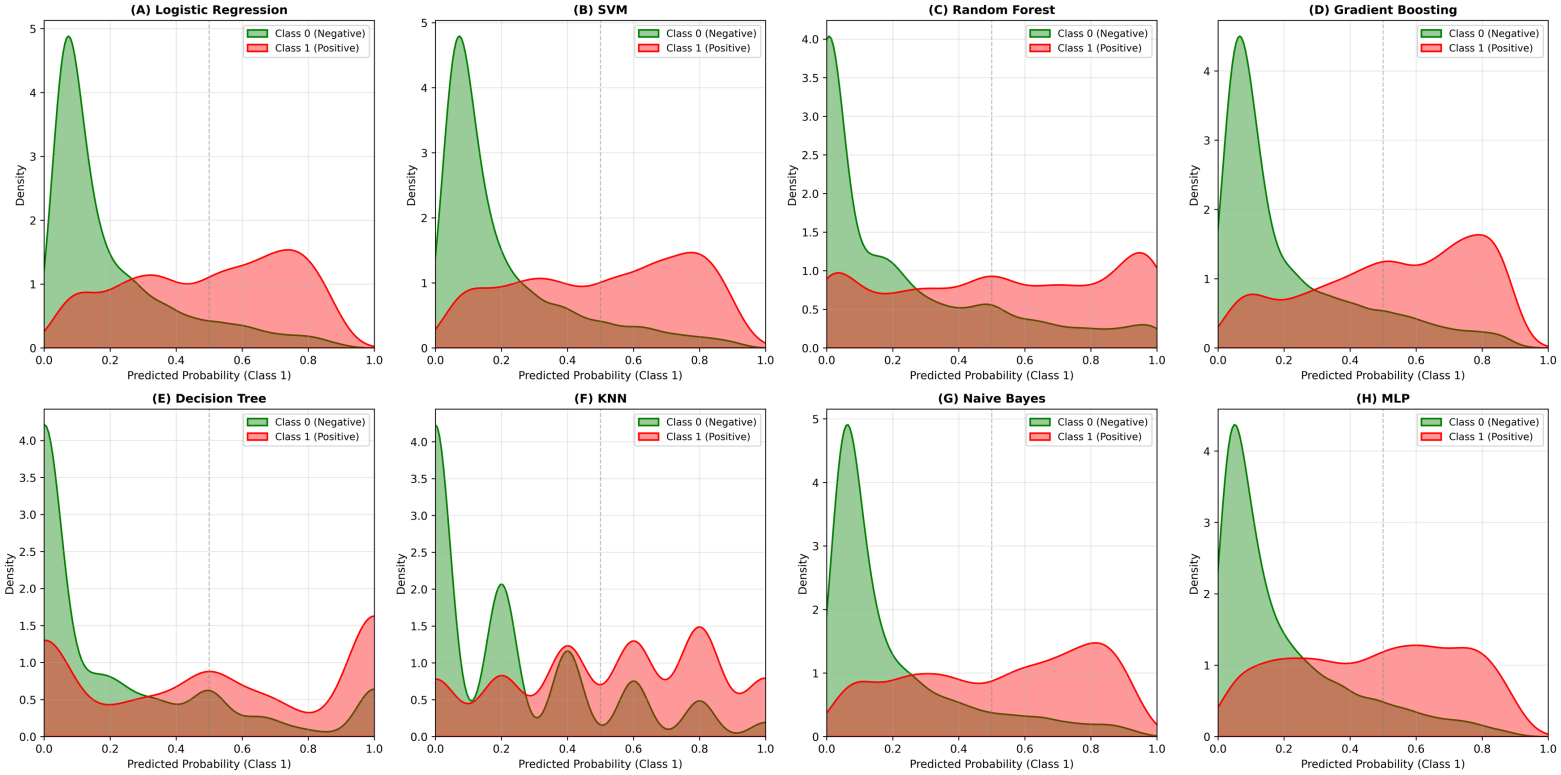
Predicted probability density distributions of lung involvement percentage by different machine learning models. Density plots show the predicted probability distributions for non-involvement (class 0, green) and lung involvement (class 1, red) across eight classifiers: Logistic Regression, SVM, Random Forest, Gradient Boosting, Decision Tree, KNN, Naive Bayes, and MLP. Models such as gradient boosting and MLP demonstrate better class separation, indicating higher discriminative performance.

**Figure 7:**
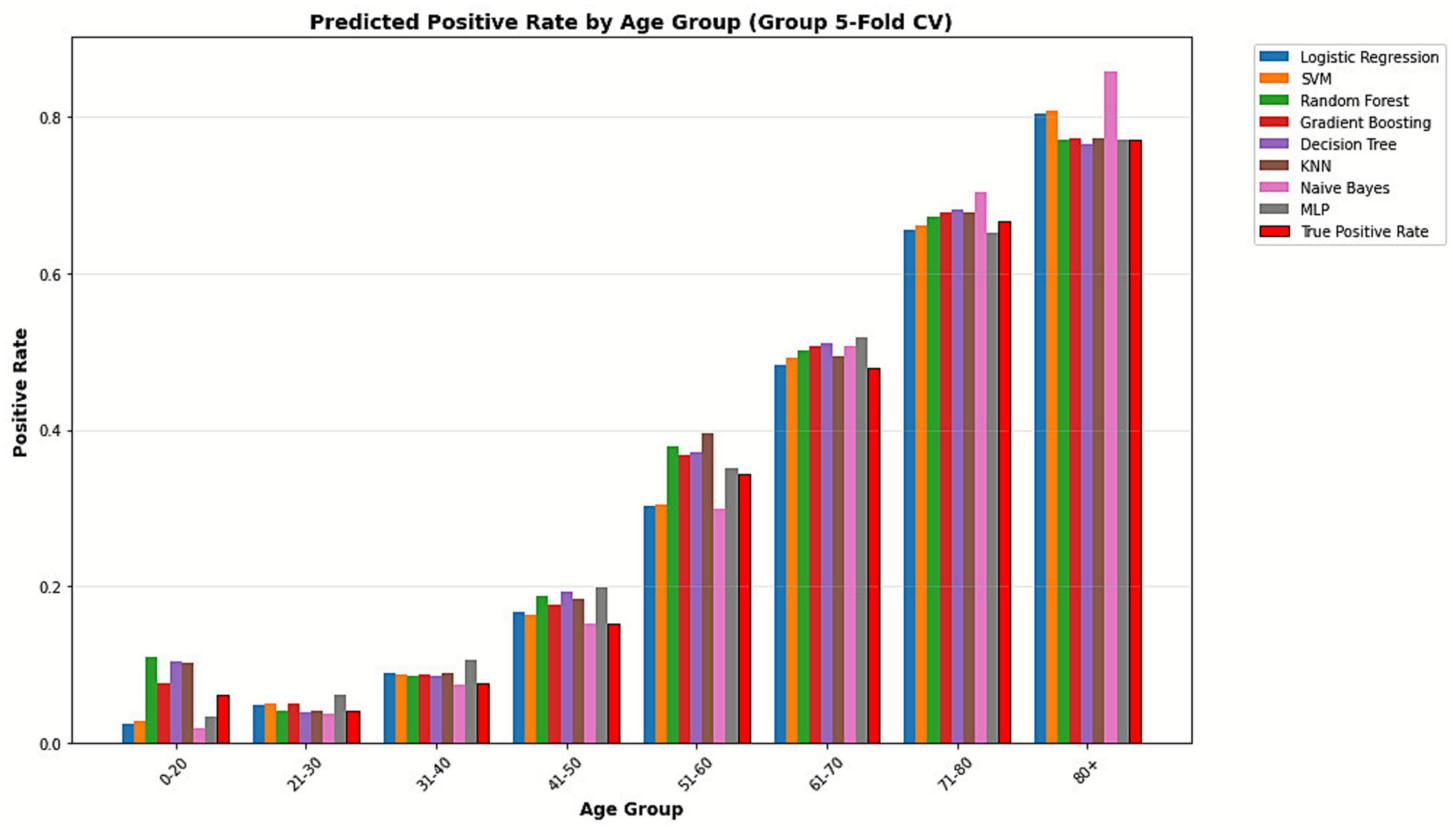
Predicted positive rate by age group across machine learning models. Bar plots show the predicted positive rates (lung involvement) across different age groups. True positive rate (red bars) increases progressively with age.

After incorporating daily case counts as an independent variable, ROC and PR curves of the models are shown in Figure 4. Confusion matrices summarizing the classification results of the eight machine learning models are presented in Figure 5, with darker colors indicating higher counts. Density plots of predicted probabilities for non-involvement (Class 0, green) and lung involvement (Class 1, red) across the eight classifiers are shown in Figure 6, illustrating the distributional separation between the two classes. Predicted positivity rates stratified by age group are shown in Figure 7. Observed positivity rates (red bars) increased progressively with age, particularly among individuals aged over 60 years, where predictions from all models showed close agreement with observed values. In younger age groups (0–40 years), greater variability in predicted positivity rates was observed across models, with lower predicted probabilities in some classifiers. In older age groups (≥50 years), predicted and observed positivity rates were generally concordant across models.

**Figure 8:**
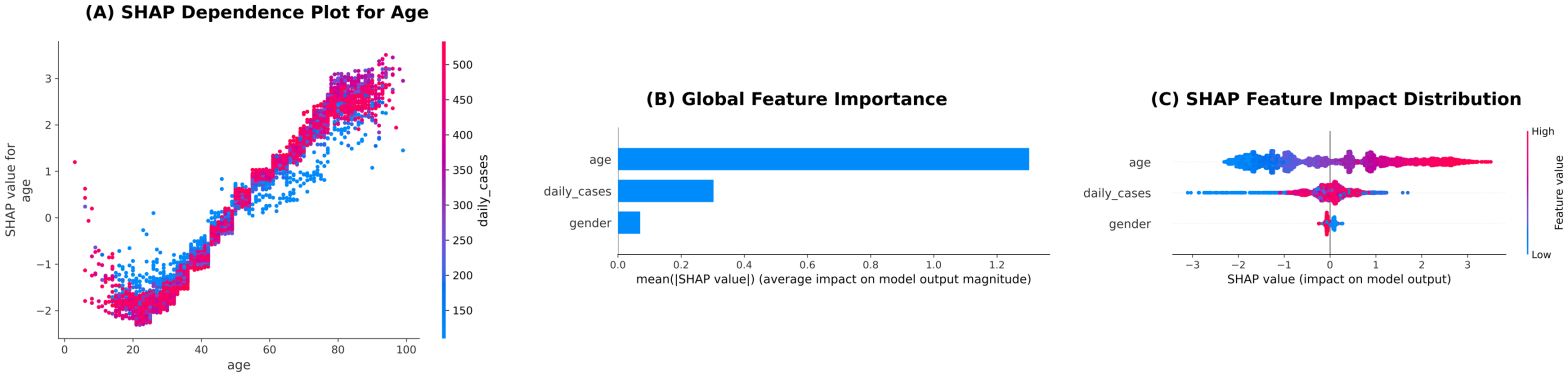
SHAP analysis of the gradient boosting model for predicting COVID-19 lung involvement percentage. **(A)** SHAP dependence plot for age showing the relationship between patient age and its impact on model prediction, with points colored by daily case counts. The spread indicates non-linear effects, particularly in middle-aged and older adults patients. **(B)** Global feature importance ranking based on mean absolute SHAP values. Age is the most influential predictor, followed by daily case counts and gender. **(C)** SHAP feature impact distribution for the top three features. Each point represents a patient, showing how feature values push predictions higher (positive SHAP values) or lower (negative SHAP values).

Using gradient boosting, the relative importance of three factors for predicting lung involvement percentage was quantified as follows: age (0.8829), daily case counts (0.1125), and sex (0.0046) (see Figure 8).

### SHAP analysis of gradient boosting model

**Figure 9:**
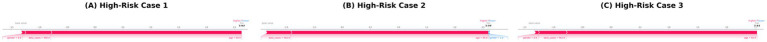
SHAP force plots illustrating model predictions for three high-risk COVID-19 cases with extensive lung involvement. **(A)** High-risk case 1 demonstrates the combined effect of advanced age and high daily case counts, both contributing positively to the elevated prediction score. **(B)** High-risk case 2 shows a 96-year-old patient where extreme age is the dominant factor driving the high-risk prediction, despite moderate daily case counts (258). **(C)** High-risk case 3 features a 93-year-old female patient with elevated daily cases (402), where both advanced age and high community transmission levels contribute to the severe outcome prediction. In all force plots: The base value represents the average model prediction. Red arrows indicate features pushing the prediction higher (increased risk), while blue arrows show features reducing risk. The final prediction f(x) is the sum of all feature associations.

**Figure 10:**
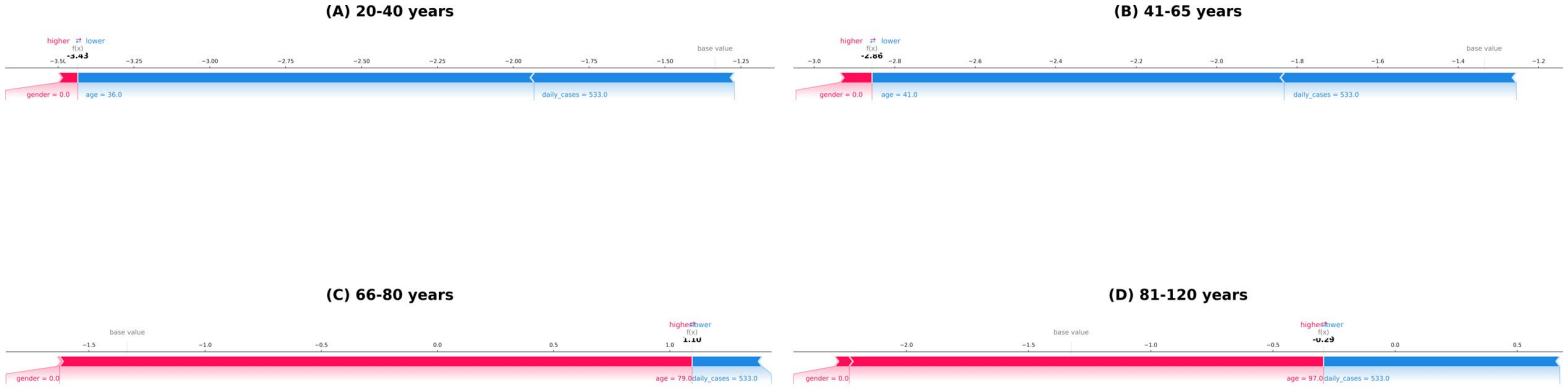
Comparison of SHAP force plots across age group with constant daily case counts (533 cases). **(A,B)** Young and middle-aged adults showing a 34-year-old and a 42-year-old patients where both age and daily cases contribute to lowering the prediction below the base value, indicating protective effects in this age group. **(C,D)** Older adults patients illustrating a 78-year-old and an 84-year-old male patients where advanced age begins to exert a stronger positive association on risk prediction. All cases share identical daily COVID-19 case counts (533), isolating the effect of age on prediction outcomes. The progression from **(A–D)** demonstrates the increasing impact of age as a risk factor for severe lung involvement.

SHAP analysis was used to examine both global and local feature contributions in the gradient boosting model (Figures 9, 10). At the global level, age showed the largest SHAP contributions, followed by daily case counts, whereas sex exhibited comparatively smaller contributions. Positive SHAP values were associated with higher age and higher daily case counts in the model-predicted lung involvement percentage, while sex showed predominantly small SHAP values.

At the individual level, SHAP force plots indicated that predictions of higher lung involvement percentage were mainly associated with advanced age and elevated daily case counts, with minimal contributions from sex. Age-stratified SHAP plots demonstrated an increasing magnitude of SHAP contributions with advancing age, with the oldest age group (≥81 years) showing the highest age-related contributions.

## Discussion

### Overview

In this study, we analyzed chest CT scans of COVID-19 patients from Suzhou, China, over a five-week period following the cessation of the dynamic zero-COVID policy, using artificial intelligence–based pneumonia analysis software. We examined associations between lung involvement and age, sex, daily case counts, and applied machine learning models with SHAP explanations. While numerous studies have investigated the effects of age and sex on COVID-19 outcomes, few have explored the impact of outbreaks, and there is a lack of research on the relationship between daily case counts and lung involvement percentage.

### CT findings

Among our Omicron cases, CT findings included ground-glass opacities, consolidation, poorly defined margins with bronchial aeration signs, with or without pleural effusion. Previous studies have reported highly variable incidence rates of these features (12). Lesions were commonly distributed bilaterally, peripherally, and in the posterior lung regions. These findings are nonspecific and overlap with other infections, limiting the diagnostic specificity of chest CT for COVID-19 (13, 14). Some literature also indicates that early CT imaging has limited value in predicting Omicron disease severity and outcomes (13). Recent studies have further suggested that overall lung involvement in Omicron infections is relatively mild (15, 16).

In our cohort, using data from patients’ first CT scans, lung lesions similarly exhibited bilateral, peripheral, and posterior distribution. Evidence suggests that COVID-19 is fundamentally an endothelial disease (17–19). From this perspective, the formation of intravascular microthrombi causing obstruction in pulmonary capillaries can manifest as ischemic necrosis in the lung interstitium (20), which aligns with radiological findings and may explain the formation of pulmonary cavities, pneumothorax, and the bilateral, peripheral, posterior distribution of lesions due to gravitational effects on microthrombi. Clinically, this mechanism may also underlie manifestations such as skin discoloration, renal impairment, stroke, and myocardial injury. Some studies indicate that chest CT can reflect not only pulmonary damage but also pathological changes associated with myocardial injury (21). Nevertheless, these interpretations remain indirect, and the extent to which quantitative lung involvement on CT represents underlying microvascular pathology warrants further investigation.

### Significant impact of age on lung involvement percentage

Our analyses consistently indicate that age is the strongest factor associated with lung involvement. GEE Logistic regression showed that older age was strongly associated with higher lung involvement percentage (OR 1.0813, 95% CI 1.0703–1.0925, *p* < 0.0001). Gradient boosting analysis confirmed this result, with age exhibiting the highest feature importance (0.8829). This finding is consistent with extensive prior literature, indicating that older patients are more likely to develop severe pulmonary damage following SARS-CoV-2 infection (22). Zhou et al. (23) also identified age as a major risk factor for severe COVID-19, with older individuals exhibiting higher risks of serious complications and mortality. Our results further corroborate these observations, highlighting that even during outbreaks without strict containment measures, age remains a critical association of lung involvement percentage.

The mechanisms underlying the impact of age on lung involvement percentage may include immunosenescence, accumulation of comorbidities, and decreased host response to viral infection. Previous studies have shown that older adults patients are more prone to cytokine storms, which exacerbate pulmonary inflammatory responses (24). The findings of our study support these mechanisms and emphasize that, particularly during uncontrolled epidemic surges, age is a key factor determining the extent of lung involvement percentage.

### Relationship between daily case counts and lung involvement percentage

Daily case counts emerged as the second most important factor. GEE Logistic regression indicated a modest but statistically significant association with lung involvement percentage (OR 1.0033, 95% CI 1.0018–1.0047, *p* < 0.0001). Gradient boosting showed that daily case counts had a feature importance of 0.1125. These findings suggest that periods of higher epidemic intensity are associated with increased lung involvement on CT. An increase in daily case counts reflects widespread viral transmission within the community. At the population level, intensified transmission has been associated with higher viral burden and increased clinical heterogeneity among infected individuals. Previous studies have reported that the extent of lung involvement in Omicron infections varies with viral load as reflected by Ct values (25), whereas lung involvement was generally minimal under strict lockdown conditions. As the intensity of viral transmission increases, the composition of infected individuals may shift toward older or more vulnerable populations, who are more susceptible to lung involvement. This epidemiological mechanism may partly explain the observed association between daily case counts and lung involvement percentage.

Another consideration is that surges in daily cases are often accompanied by increased strain on healthcare resources, potentially delaying optimal treatment and worsening patient outcomes. Wang et al. (26) demonstrated that higher hospitalization rates during outbreak periods were significantly associated with increased proportions of severe cases, likely due to healthcare systems operating under high capacity constraints. Other studies also indicate that mortality significantly rises for patients admitted during “surge” periods (27, 28). However, in our study, the Suzhou Hostpital of Integrated Traditional Chinese and Western Medicine operated with sufficient preparedness and high efficiency; we did not observe systematic delays or exclusion of patients from imaging, including those with mild disease. Therefore, the observed association between daily case counts and lung involvement is unlikely to be solely attributable to imaging delays related to healthcare system overload.

Our results indicate that although the feature importance of daily case counts is lower than that of age, it remained a meaningful factor in predicting lung involvement.

### Minimal association of sex on lung involvement percentage

Sex showed a minimal association with lung involvement percentage in our analyses. GEE Logistic regression indicated a slight protective association for females patients (OR 0.8098, 95% CI 0.6983–0.9391, *p* = 0.0053), and gradient boosting analysis yielded a feature importance of 0.0046. Indicating a minimal association on lung involvement in our study. This finding differs from some previous reports, which suggest that male patients tend to exhibit more severe symptoms and have higher rates of critical illness and mortality following SARS-CoV-2 infection (3). In our study, however, the influence of sex on lung involvement percentage was limited. This discrepancy may be related to cohort characteristics or study design. Under uncontrolled epidemic conditions, large-scale case surges may attenuate or obscure sex-related differences in pulmonary involvement across the overall patient population.

### SHAP-based interpretations

SHAP analysis provided interpretable insights into the gradient boosting model predictions, reaffirming that age was the most influential predictor of extensive lung involvement percentage, followed by daily case counts. This indicates that, according to the model, patients admitted during periods of high daily case counts were predicted to have greater lung involvement. Importantly, SHAP values reflect predictive influence within the model and do not establish formal statistical associations or causal relationships. The observed patterns likely correspond to increased viral exposure during epidemic surges rather than constraints on healthcare resources.

Furthermore, the negative SHAP values associated with female sex indicate that, within the model predictions, being female slightly reduced the predicted probability of extensive lung involvement compared with male sex. Importantly, SHAP values represent the influence of features on model outputs and do not constitute formal statistical associations or causal effects. This approach facilitates transparent interpretation of machine learning outputs in a clinically meaningful manner.

### Limitations and future directions

Although this study analyzed the primary associations of lung involvement percentage in COVID-19 using multiple machine learning models and quantified feature importance via gradient boosting, several limitations remain. Daily case counts does not directly correspond to the total number of newly diagnosed COVID-19 cases; therefore, it serves only as a proxy indicator of epidemic intensity. We only considered three independent variables—age, sex, and daily case counts—without including other potentially influential factors such as comorbidities, vaccination status, length of hospitalization, and treatment regimens. Future studies incorporating a broader range of variables could improve the predictive accuracy of the models.

Furthermore, while machine learning models such as random forests and gradient boosting perform well in capturing nonlinear relationships, they may fail to account for more complex interactions. Future research could explore more sophisticated approaches, such as deep learning models, to further enhance predictive performance.

## Conclusion

This study analyzed the associations of lung involvement percentage in COVID-19 under non-lockdown conditions and found that age was the most important predictor of lung involvement, followed by daily case counts, whereas sex had a minimal effect. Lung involvement percentage increased with both patient age and daily case numbers. Our findings provide novel insights into the clinical management of COVID-19 patients in uncontrolled epidemic settings and offer data-driven support for responding to similar outbreaks in the future.

## Data Availability

The original contributions presented in the study are included in the article/Supplementary material, further inquiries can be directed to the corresponding author.
